# Pulmonary Delivery of Antibiotics to the Lungs: Current State and Future Prospects

**DOI:** 10.3390/pharmaceutics17010111

**Published:** 2025-01-15

**Authors:** Yahya H. Dallal Bashi, Rachel Mairs, Rand Murtadha, Vicky Kett

**Affiliations:** 1 School of Pharmacy, Queen’s University Belfast, Belfast BT9 7BL, UK; rmairs06@qub.ac.uk (R.M.); rmurtadha01@qub.ac.uk (R.M.); v.kett@qub.ac.uk (V.K.); 2 College Pharmacy, University of Sharjah, Sharjah P.O. Box 27272, United Arab Emirates

**Keywords:** cystic fibrosis, bronchiectasis, inhalation, DPI, nebulizer, tobramycin, amikacin, apramycin, rifampicin, *P. aeruginosa*

## Abstract

This paper presents a comprehensive review of the current literature, clinical trials, and products approved for the delivery of antibiotics to the lungs. While there are many literature reports describing potential delivery systems, few of these have translated into marketed products. Key challenges remaining are the high doses required and, for powder formulations, the ability of the inhaler and powder combination to deliver the dose to the correct portion of the respiratory tract for maximum effect. Side effects, safety concerns, and disappointing clinical trial results remain barriers to regulatory approval. In this review, we describe some possible approaches to address these issues and highlight prospects in this area.

## 1. Introduction

Antibiotic delivery to the lungs is used for targeted treatment or management of infection in the lower airways with the rationale that inhalation offers an improved concentration at the target site, with the potential to reduce systemic side effects commonly associated with other routes of administration [1]. Often, prescribing is reserved for specific patient groups, including people with cystic fibrosis (CF), bronchiectasis, and chronic obstructive pulmonary disease (COPD). These conditions each present additional challenges that require consideration in terms of formulation, delivery device, and dosing strategies, while the presence of bacterial biofilm presents an additional barrier to delivery of antibiotics to the site of action. These factors must be considered when designing both the formulation and the mode of delivery. While there are a number of marketed products available to deliver inhaled antibiotics, mainly focused on *P. aeruginosa* infection in people with CF, there is the scope for further development of inhaled antibiotics, particularly in the management of less common pathogens, improvement in usability, and highlighting the potential to reduce systemic toxicity.

## 2. Advantages of Inhaled Delivery over Alternative Routes

In people with diseases characterized by chronic respiratory infection, antibiotic therapy is delivered orally, intravenously, or via inhalation. Inhaled preparations offer advantages over other routes as they allow a high concentration of the agent at the site of action [2,3,4]. Local antibiotic delivery also reduces systemic side effects whilst maximizing the antimicrobial effect [5]. The development of resistance could also potentially be minimized through the delivery of high concentrations at the site of action, whilst reducing the exposure to commensal bacteria at non-target sites such as the gut [6]. The potential to reduce the occurrence of antimicrobial resistance by delivering inhaled antibiotics remains controversial, with exponents pointing to lower systemic drug concentrations than those caused by less targeted oral or intravenous administration [7,8,9]. It is certain, however, that established bacterial infections present additional barriers to the effective delivery of antibiotics.

A key defense mechanism of problematic bacteria is the ability to form a biofilm, a matrix of packed communities of microorganisms. The biofilm produces an external protein, lipid, DNA, and polysaccharide matrix [10], which forms a physical barrier protecting the bacteria from antibiotics and the host immune cells [11,12,13,14,15]. Biofilm formation results in increased resistance of bacteria to antimicrobials and has been linked to treatment failure with decreased susceptibility of biofilms to antimicrobials and increased resistance to the host immune cells by up to 1000-fold [14,15,16,17,18]. Diffusion of antimicrobials through the anionic polymer matrix is inhibited by chemical and electrostatic interactions [19,20,21].

## 3. Target Patient Groups

Pulmonary bacterial infections primarily affect those with pre-existing lung diseases including CF, bronchiectasis, and COPD or a compromised immune system. CF is caused by mutations in the gene that encodes the CF transmembrane conductance regulator (CFTR), affecting the transport of chloride and other ions across epithelial cells. The resulting morbidity and mortality are generally related to lung complications [22]. The most common mutations are found in people from Europe and North America, although less common mutations are responsible for CF in people globally [23]. Despite the introduction of CFTR modulators, bacterial infection still persists for people with CF [24]. Bronchiectasis is a chronic progressive condition characterized by dilation of the airways and inflammation, which results in thickening of the bronchi walls. While the cause is not known, it is often diagnosed post-infection, such as in patients with pneumonia, and its prevalence is increasing globally [25,26,27].

### Disease-Specific Barriers to Delivery

In people with CF, mucociliary clearance is impacted, producing an increased sputum viscosity, by the presence of DNA polymers from damaged cells and apoptosis of neutrophils, along with F-actin fragments [5,28] and less water compared to healthy controls [29,30]. In bronchiectasis, there is hypertrophy of mucus-secreting glands, resulting in mucus accumulation [5,31], and problems with mucociliary clearance [5]. The difference in sputum composition between CF and bronchiectasis may affect the responses to inhaled antibiotic therapy [5]. In COPD, mucus hypersecretion causes mucociliary dysfunction. Penetration of antibiotics through mucus in the lungs in both CF and bronchiectasis is an important factor to consider when developing antibiotics as mucus can affect the bioavailability of therapeutics by acting as a physical barrier [29,32,33]. The complexity of the mucus present in chronic lung infection and its overproduction can limit drug penetration [30,34,35]. The high concentration of antibiotics achieved following inhaled therapy may eradicate organisms in sputum but must also be capable of penetrating the mucus to ensure high concentrations at the site of action in order to effectively clear the infection [5]. But inhaled drugs can become trapped in mucus and thus left unable to reach the epithelium [34], with penetration impeded by electrostatic, hydrophobic, and hydrogen-bonding interactions [36,37]. Mucus plugs containing mucins and DNA are negatively charged, so drug molecules or carriers that have a positive charge may bind to them and thus prevent penetration [37,38,39]. Furthermore, within sputum, there is a complex network of fibers formed, which prevent drug penetration, although 100–1000 nm pores may give nanoparticles the ability to penetrate them [30,35].

## 4. Bacterial Targets for Treatment

*P. aeruginosa* is the target for most of the approved inhaled antibiotics, with amikacin for non-tuberculous mycobacterial (NTM) infection an exception, as shown in Table 1. The presence of *P. aeruginosa* in all chronic lung conditions predicts a poor disease progression and worse outcomes [21,40,41,42]. Biofilm formation in *P. aeruginosa* is a major mechanism of bacterial resistance, occurring mainly due to hyperexpression of the MexCD-OprJ efflux protein comprising the membrane fusion protein MexC, the inner membrane transporter MexD, and the outer membrane channel OprJ [43].

Non-tuberculous mycobacteria NTM are commonly found in the environment and cause no harm to most people. But in those with compromised immune systems and/or pre-existing lung disease, they can cause chronic infection. NTM infection has become more prevalent in recent years [44]. The global prevalence in adults with non-CF bronchiectasis from 2018 to 2022 was estimated to be approximately 10% [45], with similar rates in CF patients in smaller studies across Europe and Australia reported more recently [46,47,48]. Several hypotheses for increasing levels of NTM exist, with greater awareness and surveillance, more accurate laboratory diagnosis, and a shift in the lung microbiota due to treatments all implicated [49,50,51].

NTM infections are notoriously difficult to treat due to the slow-growing nature of the mycobacteria, and with their location within alveolar macrophages adding a further barrier to treatment [52]. Typical treatment regimens currently last for at least one year with three or more antibiotics, resulting in problems with treatment costs and potential toxicity, and they do not routinely result in eradication of these pathogens [53,54]. Treatment options vary according to the species. Those most likely to result in lung disease are the *Mycobacterium avium* complex (MAC) and the *Mycobacterium abscessus* complex (MABSC). Chronic *P. aeruginosa* and NTM infections are often hard to treat and result in significant patient morbidity and mortality.

Additional targets for inhaled therapy include carbapenem-resistant Gram-negative bacteria, with carbapenem-resistant *Enterobacteriaceae* having been identified alongside *A. baumannii* and *P. aeruginosa* by the WHO in 2017 as priority 1 targets for new antibiotics. Carbapenem-resistant *Enterobacteriaceae* infections have risen in prevalence markedly and are associated with significant mortality, and spread is often associated with healthcare settings [55,56]. Resistance to new antibiotics can develop rapidly, while resistance to first-line treatments such as colistin is increasing [57], so inhaled drug delivery that targets new antibiotics to sites of infection should not be ignored when developing new antibiotic treatments [58].

### Bacterial Burden in People with CF or Non-CF Bronchiectasis

In children with bronchiectasis, *H. influenzae* is the most frequently isolated pathogen. In adults, 35% of patients are infected with *H. influenzae*, with *P. aeruginosa* also detected in 5–31% of patients. Other microorganisms such as *S. pneumoniae*, *S. aureus,* and *M. catarrhalis* have variable isolation rates [1]. NTM infection also occurs frequently in adults with bronchiectasis, with bronchiectasis airway damage predisposing patients to this type of infection [25]. MAC is the most common NTM infection in this patient group [25,45,59]. British Thoracic Society guidelines state that acute antibiotic therapy is used in bronchiectasis to treat exacerbations that present with acute deterioration, worsening local symptoms, and/or systemic upset [60,61]. NTM infection is becoming increasingly prevalent in older people and those with chronic respiratory conditions [62,63], and it is difficult to treat due to its resistance to many antibiotics [64,65,66,67].

## 5. Current State of the Art

Inhaled antibiotics with current regulatory approval include tobramycin, aztreonam, levofloxacin [68], and colistimethate sodium, which are indicated for the treatment of *P. aeruginosa* infection in people with CF, as listed in Table 1. These are first-line treatments for *P. aeruginosa* infection in people with CF, before intravenous therapy, with inhaled tobramycin recommended for the treatment of initial or new growth by the US CF Foundation [69]. Tobramycin acts to present the translation of bacterial mRNA into protein, aztreonam inhibits bacterial cell wall synthesis, levofloxacin inhibits bacterial DNA synthesis, while colistimethate sodium disrupts the bacterial cell membrane. Whilst they have different mechanisms of action, they each decrease the bacterial density in sputum and result in the stabilization of lung function [70]. Colistin is sometimes reserved for resistant strains of *P. aeruginosa;* however, it is also used when tobramycin is contraindicated, e.g., in hearing loss. A 2016 systematic review comparing their efficacy in people with CF did not show any significant difference in study outcomes, but that was attributed to the limited data available for comparison [71].

None of these treatments are licensed for use in bronchiectasis, and although clinical trials studying their effect in these conditions showed a decrease in *P. aeruginosa* sputum bacterial load, no improvement in exacerbation frequency or quality of life was seen [72,73,74,75]. A phase III trial, PROMIS-I, suggested a clinical benefit of colistimethate sodium delivered via inhalation in patients with bronchiectasis and *P. aeruginosa* infection, although this effect was not replicated in PROMIS-II, which was affected by the COVID-19 pandemic and prematurely terminated [76,77,78]. Amikacin is an aminoglycoside antibiotic, and an amikacin liposome inhalation suspension is approved in US, EU, and Japan for MAC lung disease in people with non-CF bronchiectasis. FDA approval was obtained through an accelerated approval pathway where the drug was shown to have an effect on a surrogate endpoint reasonably likely to predict a clinical benefit to patients.

### 5.1. Mode of Delivery

Inhaled medications are delivered via nebulizers, pressurized metered dose inhalers (pMDIs), or dry powder inhalers (DPIs). When considering inhaled delivery devices, the ideal device should be portable and easy to use, require minimal or no cleaning, deliver the optimum dose to the lungs, and be consistent in dose delivery [79]. Metered dose inhalers are small, easily portable devices but are unsuitable for antibiotic delivery as patient coordination is required for inhalation of doses and only small quantities of medication are delivered per dose (<1 mg powder per puff) [80,81]. All commercially available inhaled antibiotics, and those tested in recent clinical trials, are delivered via a dry powder inhaler or nebulizer (Table 1).

#### 5.1.1. Nebulizer Delivery

Nebulization is the most common form of inhaled antibiotic delivery. The key advantages of nebulizers are that they require a simple formulation and manufacturing methods, do not require a specific breathing pattern, and can deliver large doses of drugs, which are essential for dosing of antibiotics to the lungs. This simple approach has led to the “off-label” use of a number of intravenous antimicrobials including antibiotics and antifungals via nebulizers. For example, these are used to treat organisms such as *Acinetobacter baumannii* not covered by licensed inhaled therapies [82], or where the marketed formulation for inhalation is not available. A range of off-label inhaled treatments has been reviewed in terms of efficacy and safety elsewhere [83]; patient adherence to this type of treatment does not appear to be different to approved inhaled formulations [84]. The continued use of “off-label” antibiotics in this way indicates the gap that exists for the treatment of a wider range of bacterial infections.

The simple formulation and ease of use of nebulizers are counterbalanced by the traditional association with long administration times combined with the necessity of significant cleaning after each dose. Newer mesh nebulizers have overcome this issue with shorter administration times; however, they still require cleaning after each dose and are relatively expensive. Although technique issues are more commonly regarded as an issue with DPIs, there have been reports of issues with nebulizers too [85,86,87]. Liposomal amikacin and aztreonam are both supplied with disposable nebulizer handsets, and their higher cost compared to the other inhaled antibiotics incorporates the cost of these handsets [83].

Reports present sometimes conflicting findings on the preferences of users of nebulized medicines for one nebulizer over another, although most conclude that patient preference is important for adherence and better treatment outcomes. A systematic review of nebulizer systems in CF was recently published [88], and there are fewer reports on the attitudes of people with bronchiectasis, although a recently report paper presented a mixed-methods approach to understanding the experiences of people with BE and their carers. DPIs were regarded as preferable if they were as effective as nebulized treatments because of their ease of use and speed [87].

#### 5.1.2. DPI Device Considerations

Of the commercial antibiotics available, only two are delivered via DPIs—tobramycin and colistin. DPIs are similar to pMDIs with their small size and portability [80,89] and offer advantages over nebulized treatments because of their convenience and the decreased time required for each dose [90]. Patients with chronic lung disease may have issues with DPI inhalation as there is a requirement for a good respiratory effort whilst the inhalation technique affects lung deposition [80,81,91]. The drug loading of each capsule is often low, which means several capsules can be required per dose for antibiotics [80,81,92,93]. Additionally, DPIs have had more reported adverse events, with coughing commonly reported [81,94]. A study investigating the preferences of people with CF toward different hypothetical treatments revealed a heterogenous response; unsurprisingly, many selected hypothetical options, with an improvement in life expectancy by ≥10 years having the greatest impact on treatment preference, followed by a 15% increase in lung function. However, it was shown that people would trade substantial reductions in these key outcomes for a reduced treatment time or burden [95]. This is in broad agreement with a 2016 study that showed that people with CF would hypothetically tolerate a dry cough if treatment was switched from a 30 min nebulizer twice daily to a 10 min DPI twice daily [96].

Antibiotics require high doses, generally requiring multiple capsules to be administered, and most devices require more than one inhalation (“puff”) to completely empty a single capsule [97]. This can be inconvenient for users, who need to insert multiple capsules, so antibiotic DPIs can increase the therapy burden. DPIs do not contain propellants, so they rely on a minimum inspiratory flow rate (IFR) to disperse the powder, which will generate fine particles that will reach the correct portion of the airways. Unfortunately, the IFR is not generally determined during patient lung function tests. Different devices will exhibit different resistances to airflow, and it is important to match the device with the formulation and the user in order to ensure a good deposition, minimal powder in the mouth and throat, and minimal side effects associated with these such as dry coughing [96].

Devices can be classed as low-, medium-, or high-resistance devices according to the pressure drop across the device. Low-resistance devices exhibit a pressure drop < 5 Mbar^1/2^Lmin^−1^, high-resistance devices exhibit a pressure drop > 10 Mbar^1/2^Lmin^−1^, while medium-resistance devices are in the range of 5–10 Mbar^1/2^Lmin^−1^. The inverse relationship between device resistance and required inspiratory flow rate was described for a range of commonly used DPI devices [98]. Devices with high resistances require a lower effort, which makes it easier for the user to generate the necessary flow rate. Low-resistance devices may be difficult to use for people with a poor lung function because of the high flowrate necessary to generate the powder, which also results in high-velocity particles, with higher deposition rates in the oropharynx. For example, users of the Tobi Podhaler device, a breath-actuated inhaler with low resistance for the delivery of tobramycin, are instructed to inhale twice to ensure that all contents of the capsule are completely delivered, with a similar requirement for the colistimethate sodium Turbospin inhaler. Devices with trigger valves are useful to ensure that powder is only released once a minimum airflow has been achieved, which is particularly important for formulations with large particle sizes (3–6 micron diameter) [99]. Some devices used for other therapies include feedback mechanisms that enable the user to tell if they have correctly deployed the device (Aspire, AstraZeneca, Cambridge, UK).

Matching devices to the patient requires understanding their lung function and personal preferences; FEV_1_ is commonly used as a marker of disease severity and is often the primary outcome in assessing the lung function in clinical trials, but it is not well-correlated with the IFR. A small study in 1998 assessed the peak IFR for people with CF and people with COPD, all with a stable disease severity, across a range of resistive loads (6.5–213 × 10^3^ Pa^0.5^sm^−3^) and demonstrated that they would all be able to generate sufficient inhalation to meet the requirements of the devices available at that time, and that only the highest resistances < 68 × 10^3^ Pa^0.5^sm^−3^ generated “moderate discomfort” [100]. However, a significant challenge related to DPI efficacy is usability and its relationship with functionality, where usability is used to describe how the user interacts with the device, while functionality describes how reproducibly and efficiently powder is delivered to the correct area of the lungs. Usability is related to the device design, while functionality is dependent on both the device and the formulation.

Usability has a large impact on efficacy, with device training of key importance in determining how well the user is able receive the dose in the correct portion of the respiratory tract. Additional considerations include user acceptability and potential errors in use outside of the clinic. Regardless of whether an inhaled treatment is delivered by a DPI or a nebulizer, they are still more burdensome for the user than an oral tablet, especially if more than once-daily dosing is required. With the advent of modulator therapy, there have been many reports of people with cystic fibrosis stopping their inhaled antibiotics [101], with a recent paper describing the need for updated guidance for the management of infection in people who use CFTR modulators [102]. Generally, users report a higher adherence for once-daily over other regimes, and in interview-based studies, skipped doses were most likely caused by a change in routine or fatigue, rather than side effects [84].

### 5.2. Formulation of Inhaled Antibiotics

Nebulized formulations are generally simple saline dispersions requiring little specialized formulation or manufacturing processes, with the exception of liposomal amikacin, which is discussed further below in the section on nanoparticle formulations. Colistimethate sodium powder for DPI is packaged in hard gelatin capsules and delivered via a Turbospin inhaler [103]. Tobramycin for DPI (known as TIP) is a more complicated formulation also containing 1,2-distearoyl-sn-glycero-3-phosphocholine (DSPC) and calcium chloride. During a spray-drying process, tobramycin becomes coated in DSPC to create the spherical hollow “pulmosphere” particles. The powder product is administered via the breath-actuated Podhaler device; details of both the powder and the device are included in the results of the EVOLVE clinical trial [104].

#### Nanoparticle Formulations

Nanoparticle formulations offer the opportunity to protect drugs and improve their deliver through mucus and to the site of action. Many nanoparticle options have been described in the literature, although the only marketed nanoparticle product is a liposome. Liposomes are spherical nanometer to micrometer-sized vesicles consisting of one or more lipid bilayers surrounding an aqueous core [105]. Aside from liposomal amikacin, there are no other currently licensed inhaled liposomal antibiotic products [106], although some have successfully completed clinical trials. An amikacin liposomal inhalation dispersion consists of amikacin encapsulated in dipalmitoylphosphatidycholine and cholesterol liposomes, which are administered via nebulization once daily. The liposomal drug penetrates the sputum and exhibits a prolonged half-life compared to free non-liposomal aerosolized antibiotics [70]. An amikacin liposomal inhalation dispersion is licensed in the US for the treatment of a limited population of people with MAC infection, who have limited or no other treatment options, and is the only medication approved by the FDA for the treatment of MAC. Amikacin liposomal inhalation dispersion treatment for MAC received accelerated approval due to sputum culture conversion in the phase III CONVERT trial [107,108]. The trial reported drug-induced injury in a small minority of patients, with a small number of effects reported since gaining regulatory approval [109]. The amikacin liposomal inhalation dispersion was previously investigated for chronic *P. aeruginosa* infection in patients with CF in clinical trials. Trial outcomes showed the non-inferiority of the amikacin liposomal inhalation dispersion compared to a tobramycin solution for inhalation in phase III trials; however, more patients receiving the amikacin liposomal inhalation dispersion experienced exacerbations compared to those on tobramycin [110].

A liposomal ciprofloxacin inhalation dispersion developed by Aradigm (Apulmiq in the US, Linhaliq in the EU, and previously Pulmaquin) comprised dual-release ciprofloxacin, with a liposomal encapsulated drug alongside a free drug. Its use in patients with bronchiectasis and chronic *P. aeruginosa* infection was investigated in phase II and III clinical trials. Promising phase II trial results showed a decrease in *P. aeruginosa* bacterial density, as well as an increased time to first exacerbation, and it was well-tolerated by the patient group [111]. However, the results were not consistent between two international, phase III clinical trials—ORBIT-3 and ORBIT-4. Both showed an increased median time to next exacerbation, but it was only statistically significant in ORBIT-4, although the *P. aeruginosa* sputum density was significantly reduced in both the ORBIT-3 and ORBIT-4 trials [112]. The dispersion did not receive approval from the FDA or the EMA, with further clinical data analysis and a further phase III trial recommended, and Aradigm withdrew their application to the EMA in 2019.

A DPI formulation of ciprofloxacin to treat non-cystic fibrosis bronchiectasis saw good results in the RESPIRE I trial [113] but failed to meet the primary endpoints in the phase III clinical trial RESPIRE II (reduced time to exacerbation and reduced frequency of exacerbations). The authors noted that a stricter definition of prior exacerbations as an inclusion criterion might benefit future clinical trials [113,114]. However, its effect on *P. aeruginosa* in CF was non-inferior to that of a tobramycin inhaled solution [114,115]. Aerovanc (Savara, Langhorne, PA, USA) was an inhaled vancomycin DPI formulation developed for the treatment of MRSA in people with CF. The formulation progressed through phase I and II clinical trials, but the phase III AVAIL trial failed to demonstrate a significant improvement between the treatment and placebo arms FEV_1_ or the reduction in exacerbation frequency [116].

## 6. Barriers to Development of New Inhaled Antibiotics

### 6.1. Barriers Related to Clinical Trial Design

As highlighted above, recent antibiotic products (and indeed new antibiotic molecules such as murepavadin [117]) have advanced partly or completely through clinical trials without reaching the market. These costly investments without success have resulted in some larger pharmaceutical companies moving away from investment in this area. Furthermore, pre-clinical testing is more complicated for inhaled formulations than oral or injectable systems [118]. Some potential causes of failures in these clinical trials have been identified. The effect of patient inclusion criteria and study outcomes have been described extensively; for example, patients have not exhibited exacerbations during trials (RESPIRE I and II inhaled ciprofloxacin in bronchiectasis) [113,115,119]. FEV_1_ has often been selected as the primary study outcome, although FEV_1_ has improved in target populations over time, making improved FEV_1_ difficult to demonstrate, and does not correlate with quality of life, which itself is associated with a placebo effect [120]. Others have suggested that a reduction in exacerbations may be an easier and more relevant target, or a reduced time to the first exacerbation [113]. Recently, better patient-focused study endpoints have been recommended for pediatric patients with bronchiectasis [121]. In the study design, blinding can be difficult for inhaled products as there are often differences in taste and smell (and appearance for nebulized dispersions). Additionally, in people with CF for whom CFTR modulator treatments have been appropriate, improvements in lung function have been seen, but they have not improved underlying infection rates. Modulator therapies in patients with CF substantially reduce sputum production, which affects the ability to collect expectorated samples from patients using these therapies.

### 6.2. Regulatory Barriers

As bacteriophage treatments are not licensed in many parts of the world, treatments require specific approvals, for example, using an emergency Investigational New Drug application to the FDA, local ethics approval, and patient consent [122]. There are some case studies reported in the literature, for example, for compassionate treatment of resistant MABSC infection [123], but there are few clinical trials [124]. The WHO has begun to investigate how bacteriophage treatments can be used to control AMR and to generate evidence to support its use with regulatory bodies. At the national level, interest is also growing, with a report published by the UK Parliament describing the antimicrobial potential of bacteriophages and highlighting that the requirement for a UK-produced bacteriophage to meet GMP standards effectively requires clinicians to import bacterophages under special licenses with strict conditions [125]. Other jurisdictions have similar requirements, while in Belgium, a ‘magistral approach’ is used that does not require GMP standards, and those treatments can be imported into the UK if they meet the requirements of the Human Medicines Regulations 2012. The Belgian approach is currently under review by the European Pharmacopoeia Commission; the EMA published a regulatory perspective in 2016 that outlined the existing regulatory framework and identified challenges for regulatory approval such as identification, purity, potency, sterility, and stability [126]. The European Pharmacopoeia Bacteriophage Working Party is currently working on a general chapter describing bacteriophage potency, as a supplement to the 11th edition of the EP “Phage therapy medicinal products” (5.31), published in July 2024. Engagement of key stakeholders including manufacturers, researchers, and clinicians is required to drive forward the acceptance of bacteriophage treatments by pharmaceutical regulatory bodies. Additionally, there are reasons to be cautious, for example, regarding the immune response [127], as investigational bacteriophage therapy has resulted in antibody production that may have contributed toward treatment failure in some cases [123]. The possibility of gene transfer with host bacteria should also be investigated for any regulatory submission, as this has been implicated in having the potential to generate further AMR [128].

To date, Arikayce is the only marketed nanoparticle antibiotic formulation for inhalation; however, with many more under development, the lack of a systematic regulatory approach to nanomedicines may cause problems with commercialization or patient safety. For example, in the EU, the EMA has not defined nanomedicines, nor has it provided procedures to scrutinize manufacturing processes or properties. Manufacturers can select one of four possible regulatory routes, and harmonization is required to ensure that all nanoparticles follow the same processes for approval and subsequent regulation [129]. There are currently no off-patent generic versions (“nanosimilars”) of inhaled antibiotics to consider, but there is ample evidence for nanoparticles delivered intravenously that pharmacopeial monograph requirements are not sufficiently robust to identify differences in clinical data for products manufactured by different suppliers [130,131]. To tackle the regulatory gap, manufacturers should address additional concerns regarding their safety; here, the immune response is key, while the endotoxin content should also be considered during pre-clinical testing [131,132].

## 7. Prospects

A decade ago, there were only two inhaled antibiotics approved by the FDA—tobramycin and aztreonam—both targeting *P. aeruginosa* in people with CF, who were prescribed these to either eradicate *P. aeruginosa* early in infection or to suppress long-term infection. At that time, the unmet need was for those patients with long-term infection in clinical decline. Since then, the focus has moved to other pathogens in a wider range of patient groups, including those with NTM infection, as described earlier, *S. aureus* including MRSA, and *Burkholderiaceae*. NTM, in particular, has shown an increasing prevalence in people with underlying lung disease, which has been attributed to the increase in surveillance, reporting, and disruptions in the ecosystem of environmental mycobacteria from extreme weather events [133]. *P. aeruginosa* has remained a target for people with bronchiectasis (for example, inhaled tobramycin in the ERASE trial) [94,134,135] and ciprofloxacin [111,119]. Alternative targets for antimicrobial intervention include *H. influenzae* [27,136], *S. pneumoniae* [137], and *A. baumannii* in people with bronchiectasis, CF, or COPD [64,138,139], while carbapenem-resistant *A. baumannii, P. aeruginosa*, and *Enterobacteriaceae* are the WHO priority [140]. It is concerning that few new antibiotics have demonstrated efficacy against them in the recent literature [141], with a focus instead on combinations of existing antibiotics [142,143,144], non-small-molecule treatments, and repurposing or formatting of older antibiotics.

Non-small-molecule investigational treatments to target resistant bacteria include antimicrobial peptide mixtures, bacteriophages, and lysins, with bacteriophages and lysins particularly attracting increased research in recent years [145,146,147,148,149,150,151,152]. Bacteriophages offer the potential for effective personalized treatments, but they also present new stability challenges and regulatory bodies are still uncertain on how to deal with them, leaving a gap in the market for new treatments [145,148,149,153].

With a dearth of new antibiotics, the possibility of reformatting or repurposing older antibiotics has become attractive. Currently used drugs that might be reformatted include clofazimine, which was initially investigated for the treatment of *M. leprae* as a component of the WHO recommended triple-drug regimen [154]. In the US, it is available as capsules for oral administration but exhibits low solubility and variable absorption when administered orally (45–62%) [95,155,156]. This limits the success in treating pulmonary NTM, with an excessive distribution in adipose cells causing several adverse drug reactions, including skin discoloration, ichthyosis, and gastrointestinal upset [157,158]. The US Cystic Fibrosis Foundation and the European Cystic Fibrosis Society have suggested that 100 mg of clofazimine as a daily oral dose should be a part of the treatment regimen employed in the continuation phase for MAC lung disease, which also includes an oral macrolide, inhaled amikacin, and two additional oral antibiotics [159,160]. When used to treat NTM pulmonary disease, oral clofazimine is associated with several side effects leading to its discontinuation in 14–25% of patients [54,161,162]. As early as 1986, clofazimine was shown to be effective against *M. abscessus,* with a minimum inhibitory concentrations of 0.25–2 µg/mL [163], and its efficacy has been demonstrated against a range of isolates [164]. Intracellular accumulation of clofazimine, supported by in vitro and in vivo studies, has been shown to enhance anti-inflammatory activity by macrophages, making it a potential treatment for NTM lung disease [165,166]. Synergistic activity with amikacin and clarithromycin has also been demonstrated [164,167,168,169]. Pulmonary administration of clofazimine results in concentrations in the lungs up to four times greater than that achieved by oral administration [158] and could efficiently target NTM residing in alveolar macrophages, thus rapidly achieving therapeutic concentrations and reducing gastrointestinal adverse reactions [165].

Rifampicin, an antibiotic belonging to the rifamycin class, is administered orally or intravenously for the management of diverse bacterial infections, usually in combination due to the risk of resistance developing [170]. It has become an essential part of conventional TB therapy and the primary treatment protocol for specific NTM infections, particularly those caused by MAC [171]. However, it can cause a range of side effects including severe hepatotoxicity [172]. Its effect on drug-metabolizing enzymes in the liver can lead to reduced plasma concentrations of several other drugs, including oral contraceptives [173], anticoagulants [174], and antiretrovirals [175] necessitating close monitoring and potential dose adjustments. The combination of factors described above make the delivery of rifampicin via inhalation an attractive proposition [176,177,178].

Azithromycin is a macrolide, first approved by the FDA in 1991, mainly used for the treatment of respiratory and genitourinary infections [179]. Azithromycin has shown beneficial results in the treatment of bronchiectasis and cystic fibrosis, although no direct bacteriostatic or bactericidal activity against *P. aeruginosa* has been shown [180]; its efficacy is due to an anti-inflammatory effect at sub-inhibitory concentrations [181] in addition to its modest indirect antibacterial effect attributed to its modulation of virulence factors [94]. Azithromycin interferes with quorum sensing [180,182] and has been shown to decrease biofilm formation in azithromycin-treated cultures [183]. It is also known to accumulate in alveolar macrophages, monocytes, and leucocytes at high concentrations [184]. Macrolide antibiotics are historically associated with a high degree of cardiotoxicity, resulting in an increased rate of cardiac death; however, azithromycin is considered the least cardiotoxic, with minimal pro-arrhythmic effects [185]. High-dose azithromycin has also been linked to hepatotoxicity [186], although it is more commonly seen with other macrolides [179]. Ototoxicity with azithromycin use is rare, and the majority of reported cases show reversible hearing loss following long-term high-dose therapy for MAC infection [187]. Inhalation of azithromycin might overcome issues associated with oral delivery such as systemic side effects and low local concentrations achieved [20,21,42].

Apramycin is an aminoglycoside antibiotic, like tobramycin, currently only available as a veterinary treatment [188]. Apramycin shows good in vitro antimycobacterial activity against rapidly growing NTMs, such as *M. abscessus*, *M. massiliense*, and *M. bolletii* [189,190] and superior activity when compared with other aminoglycosides, tobramycin and amikacin, against clinical isolates of *P. aeruginosa* and *M. abscessus* [190,191]. Moreover, recent studies have demonstrated that apramycin can exhibit broad-spectrum in vitro activity against multidrug-resistant human clinical isolates, including *S. aureus*, carbapenem-resistant *Enterobacteriaceae*, *K. pneumoniae*, and *A. baumannii* [192,193,194,195], with activity demonstrated in murine models [196,197]. The safety, tolerability, and pharmacokinetics of apramycin have recently been investigated in healthy adults using a single dose via intravenous administration at a daily dose of up to 30 mg/kg, which is safe, well tolerated, and shares a pharmacokinetic profile comparable to that of gentamicin [198]. Apramycin has been proposed as a next-generation aminoglycoside with marginal cross-resistance to clinically relevant aminoglycosides [199,200]. Derivatives of apramycin are also under evaluation [201,202,203].

Combinations of drugs are commonly used to treat CF and non-CF bronchiectasis. Many examples in the literature indicate the synergistic effects of these combinations when formulated for inhalation [204,205,206,207,208]. However, as yet, none have been developed for commercialization. Other investigational combinations include antibiotics inhaled in combination with enzymes [209], mucoactive agents [210], or bacteriophages [209,211,212].

There are a number of marketed digital DPI devices that allow users to record data such as environmental triggers, frequency, and quality of inhalation doses used, with some benefits reported to patients and clinicians [213]. These datasets could be useful in understanding how a range of factors affect adherence and clinical outcomes and could also be used as training sets for machine learning approaches. Other advances using AI include devices that fit inhalers to monitor the inhalation technique and frequency of use such as Respiro (Arm). A study exploring the use of AI in a smartphone app (Breathe RM) to detect exacerbations in people with CF is currently recruiting in the UK (UK Cystic Fibrosis Trust trials tracker ID TT014107) with the intention to aid in developing a personalized approach to treatment—patients/clinicians can be alerted to a potential need to seek assistance or switch up the medication. A nebulizer with electronic monitoring capabilities has also been trialed and shown to increase adherence [214]. The potential for AI to be used to provide patient feedback, with appropriate clinician input, for these devices is clear. Key considerations should include patient preferences, data security and ethical management, costs, and equitable access by all patient groups [215,216,217].

AI has also been used to understand and predict how both the device type and formulation affect powder properties and behavior in the lungs. In vitro–in vivo correlations such as APSD and lung deposition are often poor [218], and powder testing requires significant amounts of investigational materials [219]. Researchers have shown the utility of modeling and machine learning approaches, for example, in assessing the impact of DPI grid design on powder performance, which has been investigated using computational fluid dynamics [220]. Others have developed models to understand how the lung anatomy affects powder/aerosol deposition in the lungs [221] or how the device and user profile affect the emitted dose [222]. Similarly, artificial neural networks (ANNs) were used to predict the FPF for formulations, drugs, and DPI devices, with the intention of reducing the burden on powder testing [223]. Developing validated models based on computational fluid dynamics (CFD) to predict pharmacokinetic parameters in humans from data relating to formulation properties such as APSD, anatomy, and breathing will greatly improve the pipeline of inhaled antibiotics by preventing attrition in early-stage clinical trials.

## 8. Summary

A range of inhaled antibiotic drug–device combinations has been developed to target bacterial infection in the lungs. There is the scope to extend this to further bacterial targets and improve the range of treatment options available. More antibiotics are needed, requiring incentivization for developers. Furthermore, inhaled formulations offer the possibility of using our existing armory more effectively. Access to inhaled drug products is also contingent on cost and stability, with many of the products currently available requiring refrigeration during transport and storage, which presents problems for their use in warmer climatic zones. New inhaled therapies should be developed, with the requirement for approved drugs to demonstrate a clear benefit to all patient groups that may benefit from them [224].

## Figures and Tables

**Table 1:** Approved inhaled antibiotics for treatment of respiratory infection.

Drug	Pharmacological Class	Form	Dose and Schedule	Indication
Tobramycin	Aminoglycoside antibiotic	Solution for nebulization	300 mg/5 mL Twice daily	*P. aeruginosa* in CF
Tobramycin	Aminoglycoside antibiotic	Nebulizer solution	300 mg/4 mL Twice daily	*P. aeruginosa* in CF
Tobramycin	Aminoglycoside antibiotic	Nebulizer solution	170 mg/1.7 mL Twice daily	*P. aeruginosa* in CF
Tobramycin	Aminoglycoside antibiotic	Powder in capsule	4 capsules (each 112 mg) Twice daily	*P. aeruginosa* in CF
Aztreonam (as Aztreonam lysine)	Monocyclic beta-lactam antibiotic	Powder + diluent for nebulizer + nebulizer handset	75 mg/mL; 75 mg per dose when mixed Thrice daily	*P. aeruginosa* in CF
Levofloxacin (as Levofloxacin hemihydrate)	Fluoroquinolone antibiotic	Nebulizer solution	240 mg per ampoule; 100 mg/1 mL Twice daily	*P. aeruginosa* in CF
Colistin (as Colistimethate sodium)	Polymyxin antibiotic	Powder for nebulizer solution	1,000,000 unit vial 1–2 vials 2–3 times a day Maximum 6 vials per day	*P. aeruginosa* in CF
Colistin (as Colistimethate sodium)		Powder in capsule	1,662,500 unit Twice daily	*P. aeruginosa* in CF
Amikacin (as amikacin sulfate)		Liposomal dispersion for nebulization + nebulizer handset	590 mg amikacin per vial Dose delivered to the lungs when nebulized is approximately 312 mg amikacin Once daily	MAC * in people without CF

* MAC (*Mycobacterium avium* complex).

## Abbreviations

AI	artificial intelligence
AMR	antimicrobial resistance
ANN	artificial neural network
APSD	aerodynamic particle size distribution
CF	cystic fibrosis
CFD	computational fluid dynamics
CFTR	cystic fibrosis transmembrane conductance regulator
CONVERTIII	clinical trial identifier NCT02344004
COPD	chronic obstructive pulmonary disease
pMDI	pressurized metered dose inhaler
DNA	deoxyribonucleic acid
DPI	dry powder inhaler
DSPC	1,2-distearoyl-sn-glycero-3-phosphocholine
EMA	European Medicines Agency
ERASE	clinical trial identifier NCT06093191
EU	European Union
EVOLVE	clinical trial identifier NCT00125346
FDA	U.S. Food and Drug Administration
FEV_1_	forced expiratory volume
GMP	good manufacturing practice
IFR	inspiratory flow rate
MAC	*M. avium* complex
MABSC	*M. abscessus* complex
MexC	membrane fusion protein of the MexCD-OprJ multidrug efflux complex
MexD	multidrug inner membrane transporter of the MexCD-OprJ complex
MexCD-OprJ	multidrug efflux protein expressed in *P. aeruginosa*
MRSA	methicillin-resistant *S. aureus*
NTM	non-tuberculous mycobacteria
OprJ	outer membrane channel
ORBIT-3	clinical trial identifier NCT01515007
ORBIT-4	clinical trial identifier NCT02104245
PROMIS-I	EudraCT: number 2015-002743-33
PROMIS-II	EudraCT: number 2016-004558-13
RESPIRE I	clinical trial identifier NCT01764841
TIP	tobramycin inhalation powder
WHO	World Health Organization
US	United States of America
